# Costimulation Blockade Increases Risk of Ventricular Rupture in a Preclinical Model of Transmural Myocardial Injury

**DOI:** 10.1016/j.jacbts.2026.101685

**Published:** 2026-09-04

**Authors:** Florian Sicklinger, Clara Heine, Daniel Finke, Laura M. Wienecke, Lorenz H. Lehmann, Norbert Frey, Florian Leuschner

**Affiliations:** a Department of Internal Medicine III, University Hospital Heidelberg, Heidelberg, Germany; b German Centre for Cardiovascular Research (DZHK), Partner Site Heidelberg, Heidelberg, Germany

Immune checkpoint inhibitor (ICI)-associated myocarditis is a rare but severe adverse event with high morbidity and mortality.^1^ Treatment strategies remain empirical and are largely based on broad immunosuppression.^2^ Abatacept, a cytotoxic T-lymphocyte–associated antigen 4-immunoglobulin fusion protein that inhibits cluster of differentiation 28-mediated T-cell costimulation, has emerged as a targeted immunomodulatory therapy for ICI myocarditis and other inflammatory cardiovascular conditions.^3^

**Figure 1:**
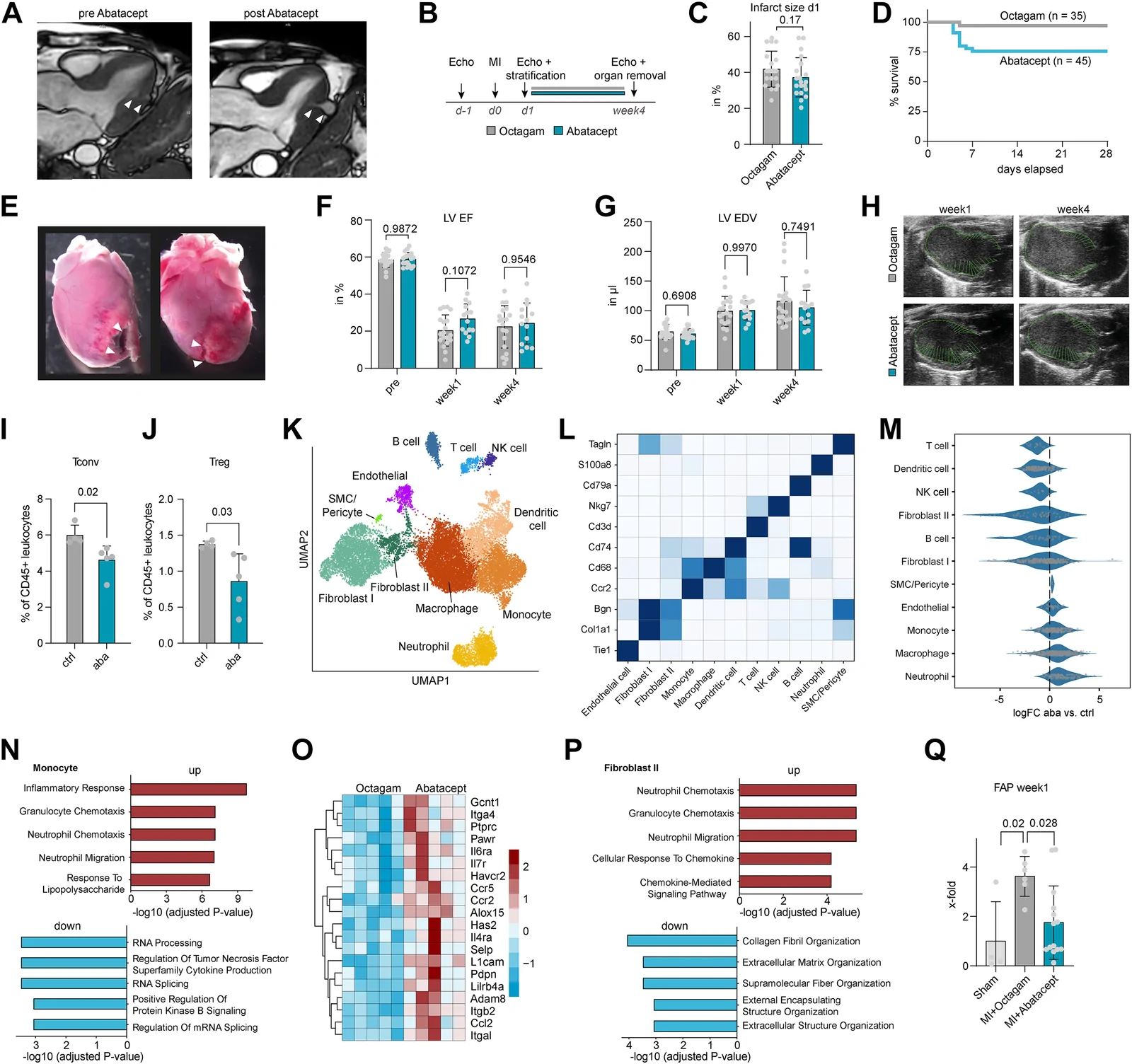
Abatacept Triggers Ventricular Ruptures After Acute Myocardial Infarction (MI) in Mice (A) Cardiac magnetic resonance imaging (MRI) images of a 67-year-old woman with immune checkpoint inhibitor (ICI) myocarditis before and after 5 cycles of abatacept treatment. (B) Experimental setup for the murine study. (C) Infarct sizes (in % of the left ventricle [LV] circumference) measured by echocardiography on day 1 post-MI (n = 20, unpaired *t*-test). (D) Survival curves for both groups (n = 35-45, log-rank test). (E) Representative images depicting left ventricular rupture in abatacept-treated animals. (F-G) Time courses of LV ejection fraction (LVEF) and LV end-diastolic volume (LVEDV; n = 14-19, 2-way analysis of variance [ANOVA] followed by Šidák's multiple-comparison test). (H) B-mode images of both groups. (I-J) Relative frequencies of circulating T-cell populations (n = 4-5, unpaired *t*-test). (K) Uniform Manifold Approximation and Projection (UMAP) embeddings of single-cell RNA sequencing data. (L) Marker gene expression. (M) Beeswarm plot showing neighborhood-level log fold-change (FC) values grouped by cell type (positive values indicate enrichment in the abatacept group; negative values indicate control enrichment, n = 2 per group). (N) Top-regulated Gene Ontology (GO) terms in the monocyte cluster. (O) Top-regulated inflammatory genes based on bulk RNA sequencing 4 days post-MI (n = 4 per group). (P) Top-regulated GO terms in the fibroblast II cluster. (Q) Myocardial mRNA expression of fibroblast activation protein (FAP) 7 days post-MI (as x-fold relative to the sham group; n = 4-14, 1-way ANOVA followed by Tukey's multiple-comparison test).

We report the case of a 67-year-old woman (institutional ethics committee approval number S-240/2017) with malignant thymic carcinoma who was treated with carboplatin and paclitaxel, followed by the ICI pembrolizumab. During ICI therapy, she developed immune-related myocarditis and generalized myositis. Because of a refractory clinical course despite high-dose corticosteroid therapy and plasmapheresis, the patient subsequently received 5 cycles of abatacept. After abatacept treatment, cardiac magnetic resonance imaging revealed a contained perforation of the left ventricle (LV) in the apical inferior region (Figure 1A). This area had previously been identified on earlier magnetic resonance imaging as one of the main lesions with transmural inflammation. Abatacept was discontinued, and the perforation was managed conservatively, with stable findings at 12-month follow-up. Frank LV perforations are rarely reported in severe ICI myocarditis. Abatacept and costimulation blockade have been previously implicated in altering the injury repair response through macrophages and fibroblasts, in addition to their direct effect on T cells. We therefore pondered whether costimulation blockade contributed to the observed LV perforation.

To investigate the effects of abatacept on cardiac repair after transmural myocardial inflammation under controlled experimental conditions, we administered abatacept in a murine model of acute myocardial infarction (MI). MI was induced in 58 male and 22 female C57BL/6N mice by permanent occlusion of the left anterior descending coronary artery under echocardiographic guidance, as described previously.^4^ Mice were intraperitoneally injected with either 200 μg cytotoxic T-lymphocyte–associated antigen 4-immunoglobulin (abatacept, Bristol Myers Squibb) or human immunoglobulin G isotype control (octagam, Octapharma) in 100 μL saline (3 times per week), with equal initial infarct sizes (Figures 1B to 1C). All animal procedures were approved by the responsible government authority (project no. G-92/21). Notably, abatacept-treated mice exhibited a higher incidence of ventricular rupture compared with immunoglobulin-treated animals (Figures 1D to 1E), with cardiac rupture confirmed as the cause of death in all deceased animals across both groups. Cardiac ruptures were restricted to male mice, mirroring the reported sex-specific vulnerability in humans post-MI. Despite increased mortality, echocardiographic analyses showed no significant differences in functional outcomes between the 2 groups at chronic stages post-MI among survivors (Figures 1F to 1H).

Because abatacept directly inhibits T-cell costimulation, we assessed the systemic T-cell response 4 days post-MI. Flow cytometry revealed significant decreases in both activated conventional T cells and circulating regulatory T cells (Figures 1I to 1J). To gain mechanistic insights into the cellular changes in the infarcted myocardium, we isolated cardiac interstitial cells 4 days post-MI by magnetic-activated cell sorting dead cell depletion and fluorescence-activated cell sorting and performed single-cell RNA sequencing. Unsupervised clustering yielded 11 distinct clusters representing major cell types of the cardiac interstitium using canonical marker genes (Figures 1K to 1L). Abatacept administration induced marked alterations in the cardiac immune-stromal landscape, characterized by increased neutrophils, monocytes, and macrophages, alongside a relative reduction in fibroblast and lymphoid populations (Figure 1M). In monocytes, Gene Ontology analysis revealed up-regulation of pathways associated with proinflammatory processes after abatacept treatment, whereas genes involved in RNA processing and RNA splicing were down-regulated (Figure 1N). Up-regulation of genes linked to proinflammatory signaling and immune recruitment was further validated by bulk RNA sequencing of LV tissue 4 days post-MI (Figure 1O). Proinflammatory signaling was also evident within fibroblasts (Figure 1P). Notably, we observed a pronounced down-regulation of genes associated with extracellular matrix organization and collagen fibril formation. Consistent with this observation, expression of fibroblast activation protein, a molecule implicated in extracellular matrix organization and scar maturation, was significantly reduced 1 week post-MI (Figure 1Q).

In summary, abatacept treatment induced a proinflammatory shift in the cardiac immune-fibroblast landscape with impaired extracellular matrix remodeling after MI. Costimulation blockade has been reported to positively influence LV function recovery after experimental MI with subsequent reperfusion,^5^ in contrast to the increased early mortality observed here. These divergent findings likely reflect context-dependent effects of immunomodulation on cardiac repair, where infarct extent and inflammatory kinetics, alongside experiment-specific factors such as genetic background and control substances, are critical determinants. In settings of acute, extensive transmural myocardial injury, costimulation blockade may impair the regulatory T-cell response, thereby disrupting the cardioprotective immune balance required for effective repair. Further studies are warranted to delineate the optimal timing and clinical scenarios in which abatacept may provide maximal benefit while preserving myocardial structural integrity.
